# Monte Carlo evaluation of tissue heterogeneities corrections in the treatment of head and neck cancer patients using stereotactic radiotherapy

**DOI:** 10.1120/jacmp.v17i2.6055

**Published:** 2016-03-08

**Authors:** Damodar Pokhrel, Christopher McClinton, Sumit Sood, Rajeev Badkul, Habeeb Saleh, Hongyu Jiang, Christopher Lominska

**Affiliations:** ^1^ The University of Kansas Cancer Center Kansas City KS USA

**Keywords:** Monte Carlo algorithm, heterogeneities corrections, head and neck cancer, re‐irradiation, SRT

## Abstract

The purpose of this study was to generate Monte Carlo computed dose distributions with the X‐ray voxel Monte Carlo (XVMC) algorithm in the treatment of head and neck cancer patients using stereotactic radiotherapy (SRT) and compare to heterogeneity corrected pencil‐beam (PB‐hete) algorithm. This study includes 10 head and neck cancer patients who underwent SRT re‐irradiation using heterogeneity corrected pencil‐beam (PB‐hete) algorithm for dose calculation. Prescription dose was 24‐40 Gy in 3‐5 fractions (treated 3‐5 fractions per week) with at least 95% of the PTV volume receiving 100% of the prescription dose. A stereotactic head and neck localization box was attached to the base of the thermoplastic mask fixation for target localization. The gross tumor volume (GTV) and organs‐at‐risk (OARs) were contoured on the 3D CT images. The planning target volume (PTV) was generated from the GTV with 0 to 5 mm uniform expansion; PTV ranged from 10.2 to 64.3 cc (average=35.0±17.5 cc). OARs were contoured on the 3D planning CT and consisted of spinal cord, brainstem, optic structures, parotids, and skin. In the BrainLab treatment planning system (TPS), clinically optimal SRT plans were generated using hybrid planning technique (combination of 3D conformal noncoplanar arcs and nonopposing static beams) for the Novalis‐Tx linear accelerator consisting of high‐definition multileaf collimators (HD‐MLCs: 2.5 mm leaf width at isocenter) and 6 MV‐SRS (1000 MU/min) beam. For the purposes of this study, treatment plans were recomputed using XVMC algorithm utilizing identical beam geometry, multileaf positions, and monitor units and compared to the corresponding clinical PB‐hete plans. The Monte Carlo calculated dose distributions show small decreases (<1.5%) in calculated dose for $D_{99},D_{\text{mean}}$, and $D_{\text{max}}$ of the PTV coverage between the two algorithms. However, the average target volume encompassed by the prescribed percent dose ($V_{p}$) was about 2.5% less with XVMC vs. PB‐hete and ranged between ‐0.1 and 7.8%. The averages for $D_{100}$ and $D_{10}$ of the GTV were lower by about 2% and ranged between ‐0.8 and 3.1%. For the spinal cord, both the maximal dose difference and the dose to 0.35 cc of the structure were higher by an average of 4.2% (ranged 1.2 to −13.6%) and 1.4% (ranged 7.5 to −11.3%), respectively, with XVMC calculation. For the brainstem, the maximal dose differences and the dose to 0.5 cc of the structure were, on average, higher by 2.4% (ranged 6.4 to −8.0%) and 3.6% (ranged 6.4 to −9.0%), respectively. For the parotids, both the mean dose and the dose to 20 cc of parotids were higher by an average of 3% (ranged ‐0.2 to −5.9%) and 4% (ranged ‐0.2 to ‐8%), respectively, with XVMC calculation. For the optic apparatus, results from both algorithms were similar. However, the mean dose to skin was 3% higher (ranged 0 to ‐6%), on average, with XVMC compared to PB‐hete, although the maximum dose to skin was 2% lower (ranged −5% to 15.5%). The results from our XVMC dose calculations for head and neck SRT patients indicate small to moderate underdosing of the tumor volume when compared to PB‐hete calculation. However, $V_{p}$ was up to 7.8% less for the lower‐neck patient with XVMC. Critical structures, such as spinal cord, brainstem, or parotids, could potentially receive higher doses when using XVMC algorithm. Given the proximity to critical structures and the smaller volumes treated with SRT in the region of the head and neck, the differences between XVMC and PB‐hete calculation methods may be of clinical interest.

PACS number(s): 87.55.K‐

## I. INTRODUCTION

With recent technological developments in linac‐based radiosurgery that employ highly reproducible patient positioning and image‐guided localization procedures, stereotactic radiotherapy (SRT) treatment has become a viable treatment option for inoperable patients with primary, recurrent, or metastatic tumors in the head and neck region.^(1)^ The heterogeneity‐corrected pencil‐beam algorithm (PB‐hete),^(2)^ which uses effective path length or superposition convolution algorithms,^(3)^ has been clinically utilized for a long period of time for computing dose calculations. However, due to the complexity of patient anatomy in the head and neck regions that includes air cavities, irregular surface curvature, dental artifacts, and bony structures, simple algorithms such as PB‐hete or convolution method^2^, ^3^ may not be robust enough to accurately predict dose distributions. More accurate algorithms may prove valuable, especially in reirradiation cases of the head and neck with hypofractionated dosing where accurate prediction of dose distribution to conformal target volumes and organs at risk is critical. Where clinically available, more accurate dose calculation algorithms, such as Monte Carlo‐based (MC) method,^4^, ^5^, ^6^, ^7^ could prove beneficial in such cases in which there exists a delicate balance between side effects and tumor control.

MC‐based algorithms have been considered a more accurate, albeit more complex, method for performing dose calculations in patient CT datasets. The improved dose calculating power stems from the algorithm's ability to accurately simulate radiation transport of secondary scatter photons and lateral electron equilibrium. As a result, MC‐based dose calculations can more accurately predict dose distributions inside complex patient anatomy including air cavities, surface irregularities, and heterogeneous tissue interfaces. In a retrospective MC study for head and neck patients by Wang et al.,^(4)^ for 6 MV beam using individual fields, MC‐calculated doses to tissues directly behind and within an air cavity were lower. After combining the fields used in each treatment plan, the overall dose distributions were similar between the two algorithms. However, MC‐computed treatment plans not only demonstrated that the target volume encompassed by the prescription isodose line was on average 2.2% lower with MC, but also that increased doses were realized for critical structures such as the spinal cord. Nevertheless, the authors concluded that both normal tissue inhomogeneity and surgical air cavities on the target coverage were adequately characterized by the conventional pencil beam algorithm when compared to MC‐based calculation. The challenges of air‐tissue interfaces were characterized by Solberg and colleagues,^(5)^ who undertook phantom measurements with and without air‐gap inserts and evaluated the ability of a MC algorithm to account for dose perturbation using a 10 MV beam. In their smaller field geometry, which used a radiosurgical setup with a 1 cm circular tissue diameter and a 3 mm air cavity at 2.6 cm depth, both film and diode measurements suggested a reduction of central axis dose by up to 21% in the area immediately following the cavity. However, the electronic equilibrium was reestablished over the next centimeter and after that the dose exceeded that of a homogenous case by up to 4%. It was observed in their experiment that MC and diode data agreed within measurement uncertainty. Thus, MC‐calculated dose has the potential to match the physically measured dose distributions.

It is expected that dose calculation algorithms in commercial treatment planning systems (TPS) must have accurate modeling to account for tissue density heterogeneity corrections in order to precisely deliver the prescribed dose to patients. Recently, several commercial TPS have implemented MC‐based dose calculation algorithms clinically that could calculate more realistic dose distributions in low‐density tissues such as lung or sinus, allowing for more accurate dose distribution in patients with lung tumors or with air cavity interfaces.^8^, ^9^, ^10^ However, dosimetric evaluation of MC‐based heterogeneity corrections for head and neck SRT patients using XVMC calculations has not been presented. In our clinic, we have implemented X‐ray Voxel Monte Carlo (XVMC) algorithm (BrainLab iPlan, version 4.1.2, Feldkirchen, Germany) for dose calculations for SBRT patients.^(10)^ Encouraged by our initial clinical implementation of XVMC algorithm, in this paper we report the dosimetric evaluation of head and neck SRT plans using XVMC as a part of validation and testing of our TPS. The main purpose of our publication is to retrospectively evaluate dosimetric parameters for target coverage and important critical structures using MC‐based methods. Our results were obtained by applying iPlan XVMC algorithm for dose calculation and dose‐volume histogram (DVH) normalization in patients who underwent head and neck reirradiation using SRT.

## II. MATERIALS AND METHODS

### A. Patient CT simulation and target contouring

A total of 10 patients who underwent head and neck reirradiation using SRT at our institution were included in this Institutional Review Board‐approved retrospective study. The original disease histology was recurrent squamous cell carcinoma for all patients. The median patient age at the time of reirradiation was 66 years (ranged 45 to 90 years). The male to female ratio was 1:1. The computed tomography (CT) simulation was performed on a 16‐slice Phillips Brilliance Big Bore CT scanner (Philips Healthcare, Andover, MA). During the CT simulation scan, patients were immobilized in the supine position with arms on the chest holding a blue ring and legs supported by a knee roll. The patient's head was immobilized using a thermoplastic mask which was fixed at the base to a stereotactic head and neck localization box (Brainlab AG, Feldkirchen, Germany). The Brainlab head and neck localizer is mounted on Brainlab head and neck system. This localization box consists of a Z‐shaped fiducial line that is filled with aluminum strips with low CT density on each side of the box. The fiducial lines produce small points in each axial CT slice and serve as a stereotactic localizer for the patient setup and verification. The 3D CT images were acquired with 512×512 pixels at 0.7 mm slice thickness and 0.7 mm slice spacing. All DICOM 3D CT datasets were then electronically transferred to the Brainlab iPlan TPS for target volume, gross tumor volume (GTV), and OARs contouring purposes. Target volume and the OARs were delineated by an experienced radiation oncologist on the T1/T2‐weighted MRI images which had been registered to the planning CT images. In some cases, a corresponding PET scan was fused to the simulation CT scan in order to aid with delineation of the target volume. In general, the planning target volume (PTV) was generated with 0 to 5 mm uniform expansion around the GTV with tighter margins used near critical structures or in the buildup region. The PTV ranged from 10.2 to 64.3 cc (average=35.0±17.5 cc). The OARs were delineated on the 3D planning CT and consisted of: brainstem, spinal cord, optic apparatus (optic chiasm and bilateral optic nerves), parotids, and skin. The skin structure was created as a 5 mm margin inside the external body contour.

### B. Clinical SRT planning process

**Figure 1:**
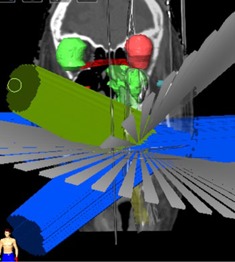
Demonstration of a hybrid arrangement of noncoplanar conformal arcs and static beams for a head and neck cancer patient treated with SRT. 3D views of left and right eyes, brainstem (green) and spinal cord (yellow) are shown with respect to the beam geometry.

Clinically optimal SRT treatment plans were generated as a hybrid plan utilizing a combination of 3D conformal noncoplanar arcs and nonopposing static beams for the Novalis‐Tx linear accelerator (Varian Medical Systems, Palo Alto, CA) with a Brainlab system consisting of high‐definition multileaf collimators (HD‐MLCs: 2.5 mm leaf width at isocenter) and 6 MV‐SRS (1,000 MU/min) mode (see Fig. 1). No additional margin for dose buildup was applied at the edges of the MLC blocks beyond the PTV. All treatment plans were calculated using PB‐hete algorithm for heterogeneity corrections with $2.0\times 2.0\times 2.0\;{\text{mm}}^{3}$ dose grid sizes. The PB‐hete algorithm in iPlan corrects for tissue inhomogeneity by computing the equivalent path length along each ray direction. It also takes into account secondary photons and electrons up to certain threshold energy, MLC design (rounded leaf and tongue‐and‐groove effect), and source function corrections such as finite source size, collimator, and flattening filter scatter. Detailed information on the PB‐hete algorithm and its clinical implementation can be found in Brainlab Technical Reference Guide.^(10)^ These patients were treated with doses of 24‐40 Gy in 3‐5 fractions with at least 95% of the PTV receiving 100% of the prescription dose. Figure 1 shows the head and neck SRT treatment planning setup.

For the head and neck SRT treatment delivery, initial repositioning of the patient was achieved by using the stereotactic localization box on the treatment couch. A pair of oblique kilovoltage X‐ray images was acquired and automatic 2D‐to‐3D image registration was performed in the ExacTrac system. This was followed by onboard cone‐beam CT scanning for further improvement of target localization. Before delivering each SRT treatment, a daily quality assurance check on kilovoltage to megavoltage imaging isocenter coincidence was performed, including Winston‐Lutz test for precise and accurate target localization. All the quality assurance procedures were in compliance for SRT treatment delivery.

### C. XVMC algorithm and clinical validation

We recently commissioned and clinically implemented the XVMC algorithm in the Brainlab iPlan RT (version 4.1.2) TPS at our institution. During the commissioning process of XVMC algorithm, all parameters were written into a dose profile file. This file was then linked to the machine profile of the Novalis Tx linear accelerator. The same file was linked to the dose profile for the PB algorithm. This means the XVMC algorithm cannot be used without the PB algorithm. Using the identical beam geometry, field sizes, and depths; same number of monitor units was delivered in Solid Water phantom to validate these algorithms. The XVMC was based on the X‐ray Voxel Monte Carlo algorithm^8^, ^9^ that consists of source modeling, beam collimating system modeling, and patient dose computation. The dose calculation parameters for XVMC in iPlan are spatial resolution, mean variance, dose result type, and MLC model. The spatial resolution defines the size of the dose calculation grid whereas the mean variance estimates the statistical uncertainty of the MC dose calculations. Choosing smaller mean variance yields more accurate dose calculations at the cost of increased computation time. Dose type can either be selected for dose to water or dose to medium. We have selected dose to the medium. Readers are advised to refer to the Brainlab Technical Reference Guide^(10)^ for more details for XVMC algorithm and its clinical implementation.

There are several studies reported in the literature on validation of XVMC dose calculations in both homogenous and heterogeneous media utilizing the iPlan RT Dose planning system.^11^, ^12^, ^13^, ^14^ In recent studies by Sethi and colleagues,^(11)^ XVMC algorithm‐based calculation was scaled using ion chamber and EDR films in various depth in five different phantoms with four different density materials irradiated with 6 MV photons. These materials included tissue‐equivalent plastic water, lung‐equivalent (low and high density) corks, and bone tissue. For heterogeneous lung phantoms, there was excellent agreement (less than 3% difference) between measured and calculated dose profiles with XVMC. Compared to XVMC, PB‐hete calculations overestimated mean PTV measured dose by up to 34%. The measured versus PB‐hete calculated dose difference increased with decreasing field size, decreasing density, and increasing depth within heterogeneous medium. However, similar results were observed beyond heterogeneous tissue. In contrast to the overestimation of mean PTV dose, large underestimation of dose (up to 50%) was observed in the penumbra region while using the PB‐hete algorithm.^(11)^ In the validation study by Petoukhova et al.,^(14)^ the XVMC algorithm‐based dose calculation was observed to agree with ion chamber and film measurements using an Alderson anthropomorphic phantom with both measured and calculated clinical 6 MV photon plans on a Novalis linear accelerator. For fields larger than the dimensions of the inhomogeneity, the XVMC calculated dose was within 3%/1 mm agreement with the measured data for the inhomogeneous phantom with lung, lung/bone equivalent material, and air cavities. However, for small field sizes, some deviations were found within and a few mm behind the air cavities. Dosimetric analysis for 10 lung cancer patients showed a difference of up to 22% within the target volume. This difference was smaller, however, for the five head and neck cancer patients who were evaluated.

Our own initial clinical experience^15^, ^16^, ^17^ on validating and implementing iPlan XVMC algorithm using QUASAR (Modus Medical Devices Inc., London, Canada) phantom study has demonstrated an excellent agreement (within ±2%) between doses calculated using XVMC versus ion chamber measurements for 6 MV‐SRS beams in heterogeneous lung equivalent material. In our phantom study,^(15)^ the dose difference between PB‐hete and measured value was as large as 9%. XVMC algorithm accurately predicted both the dose delivered to the isocenter and the dose at the boundaries of tumors whereas PB‐hete overestimated the dose at the lung‐tumor interfaces due to the lack of electronic equilibrium in the regions near low‐density tissues and heterogeneous interfaces. In our most recent clinical study^16^, ^17^ using a large cohort of lung SBRT patients, the mean PTV dose was overestimated by 15%, on average, when using PB‐hete algorithm compared to XVMC using identical beam configurations, MLCs margins, and total number of MUs. Also, the volume of lung receiving 5 Gy, 10 Gy, and 20 Gy was overestimated by about 3.0%, on average, when calculated by PB‐hete compared to XVMC. Additionally,

we observed that the dose calculation accuracy is also dependent on tumor size and location. Our results were consistent with the previously reported literature.^11^, ^12^, ^13^, ^14^ The peer‐reviewed literature has documented the robust experimental validation and clinical implementation of the XVMC algorithm to accurately predict dose delivered to heterogeneous tissue interfaces.^11^, ^12^, ^13^, ^14^, ^15^, ^16^, ^17^ Inspired by our initial clinical experience in Monte Carlo‐based lung SBRT planning,^15^, ^16^, ^17^ we extended the use of XVMC algorithm to tumors in the head and neck regions. The goal of this article is to present comparative dosimetric analysis of our MC‐based dose calculation for the head and neck SRT patients as a part of clinical validation of a complex, yet, more accurate algorithm in real patients who underwent head and neck reirradiation. We have evaluated target coverage and the dose to critical structures such as spinal cord, brainstem, and optic apparatus, as well as skin.

### D. XVMC dose calculations

Using the same beam geometry, PTV to MLC margin, and number of MUs, plans were recomputed using the XVMC algorithm and then compared to the corresponding PB‐hete plan. All treatment plans were calculated using XVMC algorithm for heterogeneity corrections with identical dose grid sizes $\left(2.0\times 2.0\times 2.0\;{\text{mm}}^{3}\right)$ as in PB‐hete, along with 2% variance (relative standard deviation of the mean), dose to medium and accuracy optimized for MLC modeling. The 2% variance is normalized per beam; the final variance in the target volume could be smaller. However, in the nonoverlapping regions it remains 2% (i.e., the average dose at that dose point). The same prescription dose, number of fractions and isodose lines were chosen as in the clinical PB‐hete plans for plan comparison.

### E. Comparison of PB‐hete and XVMC plans

Due to the difference in prescription dose for different patients, the comparison between the PB‐hete and XVMC calculations was carried out as relative percentage dose differences in terms of mean, standard deviation (SD), and range values for the target coverage and OAR doses. The percentage dose differences for $D_{99}$, mean and maximum doses, and the target volume encompassed by the prescribed percent dose ($V_{p}$) were evaluated for coverage of the PTV. For the GTV, both $D_{100}$ and $D_{10}$ were compared between the two algorithms. For the given prescription dose, the percentage dose differences between two algorithms was evaluated for OAR doses including: spinal cord (maximum and 0.35 cc), brainstem (maximum and 0.5 cc), and optic apparatus (maximum and 0.2 cc). In addition, mean and maximum dose differences for the skin and mean dose and dose to 20 cc of parotids were analyzed. Statistical analysis was performed using Microsoft Excel (Microsoft Corp., Redmond, WA). The mean and standard deviation values for each of the dose metrics were compared using two‐tailed paired *t*‐tests for PB‐hete vs. XVMC computed dosimetric parameters for the target coverage and the OARs doses using an upper bound of p−value<0.05.

**Figure 2:**
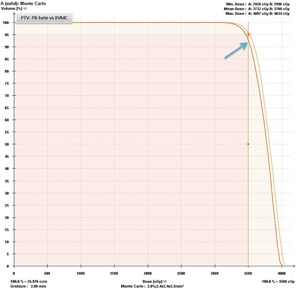
Dose‐volume histograms comparing the PTV coverage predicted by XVMC (solid line) and PB‐hete (dashed line) for Patient #6. The PB‐hete PTV coverage demonstrates at least 95% of the PTV is covered by the prescription dose of 35 Gy with a mean PTV coverage of 37.8 Gy, and a maximum dose of 40.3 Gy. However, the XVMC computed mean PTV coverage was lower by about 2% or less compared to PB‐hete. In this case, PB‐hete overpredicted $V_{p}$ by nearly 4% (light blue arrow on the DVH) compared to XVMC, leading to potential underdosing of the tumor volume. This is due to the underlying characteristic behavior of the XVMC algorithm that more accurately predicts dose distributions in surrounding low density heterogeneous tumor interfaces. The underlying characteristics of XVMC could be explained by the ability to more accurately account for transport of secondary scatter photons and lateral electron equilibrium, specifically, at air cavities or bone and tumor interfaces.

**Figure 3:**
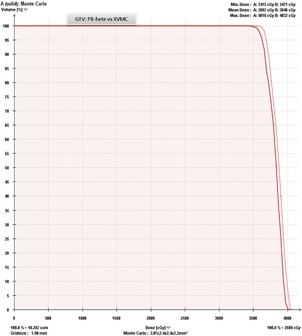
Dose‐volume histogram comparing the GTV coverage predicted by XVMC (solid line) and PB‐hete (dashed line) for Patient #6. The PB‐hete GTV coverage demonstrates almost 100% of the GTV is covered by the prescription dose of 35 Gy with a mean GTV coverage of 38.5 Gy and a maximum dose of 40.3 Gy. On the other hand, the XVMC computed values for $D_{100}\%$ and $D_{10}\%$ were lower by about 1.7% and 2% when compared to PB‐hete algorithm.

**Figure 4:**
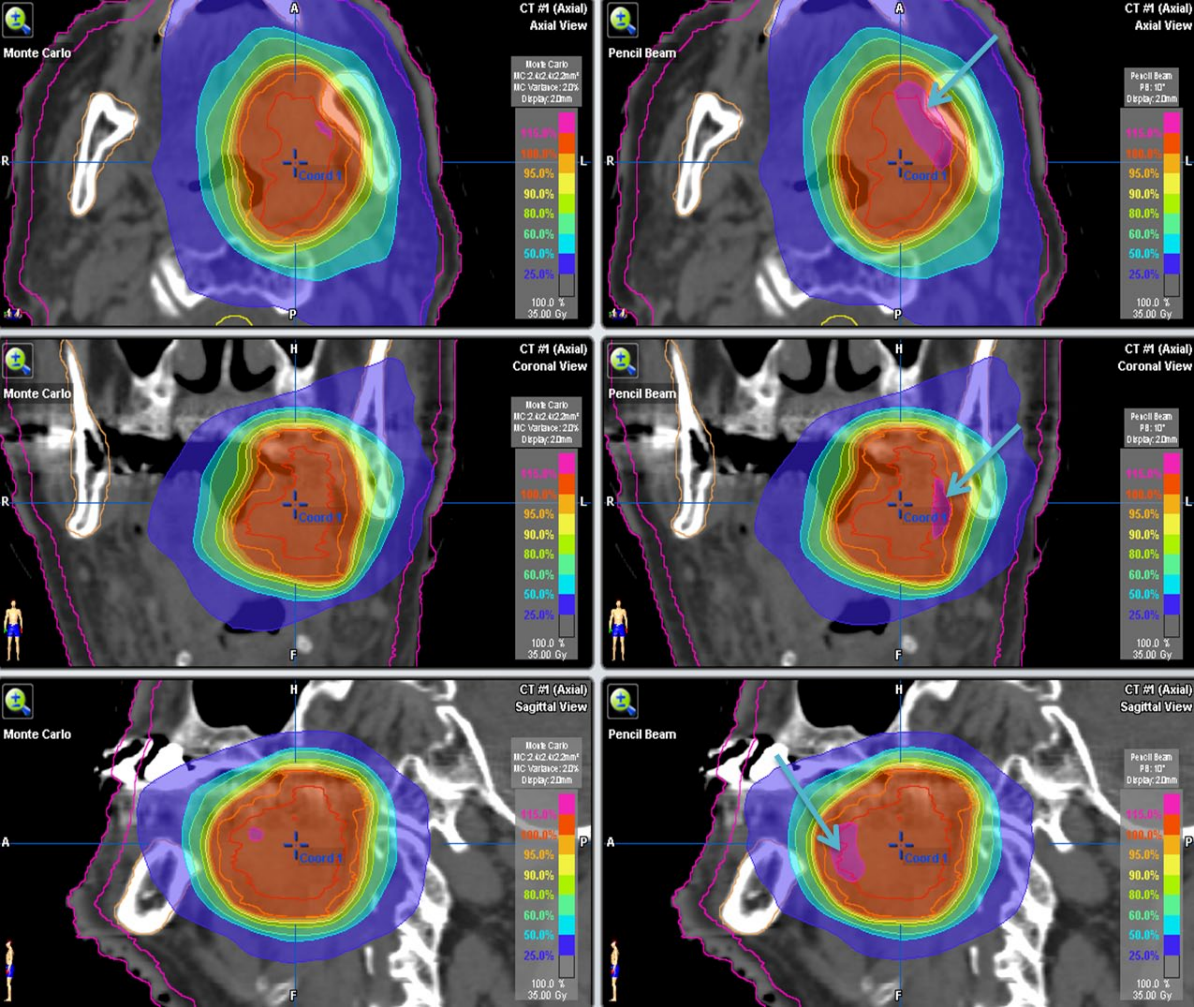
Comparison of the isodose distributions generated via XVMC (left panel) and PB‐hete (right panel) on representative axial (top), coronal (middle), and sagittal (bottom) views for a head and neck cancer patient treated with SRT. Target volumes contoured include GTV (red, innermost) and PTV (orange, outermost). Higher isodose lines, such as 35 Gy (100%), 33.25 Gy (95%), 31.50 Gy (90%), exhibit sharp dose falloff. Light blue arrows on the PB‐hete dose distributions (right panel) clearly indicate the overprediction of the PTV coverage (larger volume of 115% hotspot magenta “blob”) compared to XVMC. Other OARs such as spinal cord, mandible, and skin contours are also shown in the 3D image views.

A clinical PB‐hete computed DVH analyzing the PTV coverage for one representative head and neck SRT patient compared with XVMC calculation is shown in Fig. 2. In Fig. 3, a similar comparison is shown for the GTV coverage. PB‐hete and XVMC dose distributions observed in the axial, coronal and sagittal views for the same patient are demonstrated in Fig. 4.

## III. RESULTS

Table 1 lists the percentage differences in the dose distribution parameters for the PTV coverage averaged over 10 head and neck SRT patients. The average differences in the mean dose and associated standard deviation for $D_{99},D_{\text{mean}}$ and $D_{\text{max}}$ were all below 2% (2.1 SD) and ranged within 5%; this is due to shortcoming of the PB‐hete algorithm. However, the Vp was overpredicted by PB‐hete more than 2.0% (2.4 SD) and ranged between −0.01% and 7.7%. The average ratio of theses dose parameters were derived from the ratios for each patient and associated p‐values. $D_{99}$ and $D_{\text{max}}$ had ratios close to unity and no statistically significant p‐values were observed. However, $D_{\text{mean}}$ and $V_{p}$ achieved statistical significance with average ratios of 0.986 (p=0.001) and 0.977 (p=0.009), respectively.

Table 2 lists the percentage differences in the dose distributions parameters for the GTV $D_{100}$ and $D_{10}$ averaged over 10 head and neck SRT patients. The average difference to the mean dose and associated standard deviation for $D_{100}$ and $D_{10}$ were within 2% (1.5 SD) and ranged between −0.8% and 3.1%. The average ratio of these dose parameters were derived from the ratios for each patient and associated p‐values. However, both and reached statistical significance with ratios of 0.986 (p=0.025) and 0.987 (p=0.001), respectively.

**Figure 5:**
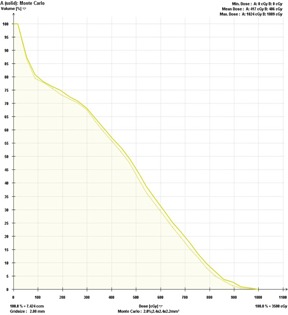
A comparison of the DVH for the spinal cord for Patient #6 computed by XVMC (solid line) and PB‐hete (dashed line). The maximum point dose and dose to 0.35 cc of the spinal cord were 1.5% and 1.8% (higher) with XVMC compared to PB‐hete algorithm.

**Figure 6:**
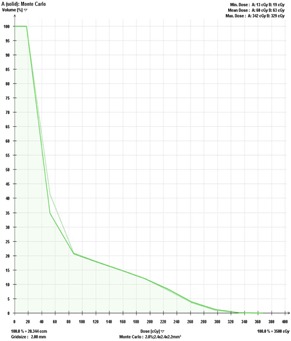
A comparison of the DVH for the brainstem for Patient #6 computed by XVMC (solid line) and PB‐hete (dashed line). The maximum point dose and dose to 0.5 cc of brainstem were 3.9% and 5.5% (higher) with XVMC compared to PB‐hete algorithm.

A comparison of the DVH for the spinal cord for Patient #6 computed by both XVMC and PB‐hete is shown in Fig. 5, while Fig. 6 shows a comparison of the DVH for the brainstem for Patient #6 computed by XVMC and PB‐hete.

**Table 1:** Percentage differences between PB‐hete and XVMC plans for the major dose distribution parameters of the PTV coverage (n=10 pts)

*Statistics*	$D_{99}$ *(%)*	$D_{mean}$ *(%)*	$D_{max}$ *(%)*	$V_{p}$ *(%)*
Mean±SD	0.8±2.1	0.8±0.6	0.2±0.9	2.4±2.4
Range	‐1.6 to 4.7	0.7 to 2.4	‐1.9 to 1.4	‐0.1 to 7.8
Ratio^a^	0.993±0.02	0.986±0.01	0.998±0.01	0.977±0.02
p‐value	p=0.24	p=0.001 ^b^	p=0.32	p=0.009 ^b^

a ^a^ XVMC/PB‐hete

b ^b^ Statistically significant p‐values.

Percentage difference=((PB−hete−XVMC)/XVMC)×100%. The negative sign indicates that the results of the XVMC plan are larger than those of PB‐hete plans.

$D_{99}\%=$ dose received by 99% of the PTV; $D_{\text{mean}}(\%)=$ mean dose received 100% of the PTV; $D_{\text{max}}(\%)=$ maximum point dose received by the PTV; $V_{p}=$ percentage volume receiving at least the prescribed percent dose; SD=standard deviation; pts=patients.

**Table 2:** Percentage differences between PB‐hete and XVMC plans for the major dose distribution parameters of the GTV (n=10 pts)

*Statistics*	*D_100_(%)*	*D_10_ (%)*
Mean±SD	1.4±1.5	1.3±0.6
Range	‐0.8 to 3.1	0.2 to 2.4
Ratio^a^	0.986±0.02	0.987±0.01
*p*‐value	p=0.025 ^b^	p=0.001 ^b^

a ^a^ XVMC/PB‐hete

b ^b^ Statistically significant p‐values.

c Percentage difference=((PB−hete−XVMC)/XVMC)×100%. The negative sign indicates that the results of the XVMC plan are larger than those of PB‐hete plans.

d $D_{99}\%=$ dose received by 99% of the PTV; $D_{\text{mean}}\%=$ mean dose received 100% of the PTV; $D_{\text{max}}\%=$ maximum point dose received by the PTV; $V_{p}=$ percentage volume receiving at least the prescribed percent dose; SD=standard deviation;pts=patients.

Table 3 lists the percentage differences in the dose distributions parameters for the OARs, including spinal cord, averaged over the 10 patients in this review. The average difference to the maximal spinal cord dose and associated standard deviation was as large as −4.2% (4.1 SD) and ranged from 1.2% to −13.6%. The average dose to 0.35 cc of spinal cord was −1.4% (5.1 SD) and ranged from 7.5% to −11.3%. The average ratio of maximal spinal cord dose was much larger than unity (1.046) with associated p−value=0.015. However, it was closer to unity for the dose to 0.35 cc of spinal cord (p=0.225). Similarly, the average difference to the maximal brainstem dose and associated standard deviation was about −2.4% (4.2 SD) and ranged from 6.4% to −8.0% and the average dose to 0.5 cc of brainstem was −3.6% (5.1 SD) and ranged from 6.4% to −9.0%. The average ratio of maximal brainstem dose was larger than unity with a value of 1.026 (p=0.019). The average ratio for dose to 0.5 cc of brainstem was 1.039 (p=0.198). Although the optic apparatus percentage dose differences were about ±2.0% (with larger SD of up to 13.2%) and ranged from 19.3% to −22.4%, the absolute dose differences were of the order of few cGy. Therefore, we do not predict the difference would be clinically significant (p=0.873, maximal optic apparatus, and p=0.863, dose to 0.2 cc of optic apparatus).

In addition to critical structures such as spinal cord, brainstem, and optic apparatus doses differences, we also evaluated mean and maximum doses to skin and mean dose and dose to 20 cc of parotids using two algorithms. The mean dose to skin was, on average, 3% higher with XVMC (ranged 0.0% to ‐6%, p=0.005), showing a statistically significant difference. However, the maximum dose to skin was, on average, 2% lower with XVMC (ranged −5% to 15.5%, p=0.233). The average values of the mean dose and the dose to 20 cc of parotids were both higher by 3% (ranged −0.2% to −5.9%, p=0.001) and 4% (ranged −0.2% to ‐8%, p=0.004), respectively, with XVMC compared to PB‐hete plan.

**Table 3:** Percentage differences between PB‐hete and XVMC plans for the major dose distribution parameters of the OARs (n=10 pts)

*OARs*	*Parameters*	Mean±SD	*Range*	*Ratio* ^a^	*p‐value*
Spinal cord	$D_{\text{max}}(\%)$	−4.2±4.1	1.2 to ‐3.6	1.046±0.05	p=0.015 ^b^
	$D_{0.35\text{cc}}(\%)$	−1.4±5.1	7.5 to ‐11.3	1.016±0.06	p=0.225
Brainstem	$D_{\text{max}}(\%)$	−2.4±4.2	6.4 to ‐8.0	1.026±0.04	p=0.019 ^b^
	$D_{0.5\text{cc}}(\%)$	−3.6±5.1	6.4 to ‐9.0	1.039±0.05	p=0.198
Optic apparatus	$D_{\text{max}}(\%)$	2.4±12.0	19.4 to ‐15.8	0.990±0.12	p=0.873
	$D_{0.2\text{cc}}(\%)$	−1.0±13.2	19.3 to ‐22.4	1.027±0.14	p=0.863

a ^a^ XVMC/PB‐hete

b ^b^ Statistically significant p‐values.

c Percentage difference=((PB−hete−XVMC)/XVMC)×100%. The negative sign indicates that the results of the XVMC plan are larger than those of PB‐hete plans. The greatest difference was seen in the case of a patient who had a large PTV volume (64.3cc) adjacent to OARs. In this case, maximum doses to the spinal cord, brainstem, and optic structures were 15.1 Gy vs. 14.1 Gy; 16.8 Gy vs. 16.4 Gy; and 4.8 Gy vs. 4.2 Gy for XVMC and PB‐hete, respectively. $D_{0.35\text{cc}}=$ dose received by 0.35 cc of spinal cord; $D_{0.5\text{cc}}=$ dose received by 0.5 cc of brainstem; $D_{0.2\text{cc}}=$ dose received by 0.2cc of optic apparatus; $D_{\text{max}}=$ maximum point dose received by the OARs; SD=standard deviation;pts=patients.

## IV. DISCUSSION

In this study, we have presented the comparative dosimetric evaluation of Monte Carlo‐based dose calculation for head and neck cancer patients treated with SRT. It was evident from our study that there is a possibility of underdosing the target volume and overdosing OARs while using PB‐hete algorithm, thus leading to a potential decrease in clinically observed therapeutic ratio. The major difference was observed in the $V_{p}$, indicating that the difference was 2.4%±2.4%, on average, and maximum up to 7.8% of the target volume that did not receive prescribed dose. These results may be explained by the underlying characteristic behavior of the XVMC algorithm that more accurately predicts dose distributions in surrounding low‐density heterogeneous tumor interfaces when compared to the PB‐hete algorithm. The ability of XVMC to more accurately account for transport of secondary scatter photons and lateral electron equilibrium (specifically, at the air cavities or bone and tumor interfaces) may likely explain the discrepancy in calculated dose distribution between the two algorithms. On the other hand, the maximum dose to critical structures such as spinal cord and brainstem were underestimated using PB‐hete algorithm. Overpredicting target coverage and underestimating dose to OARs could have critical implications, particularly in cases of head and neck reirradiation when SRT is employed. In addition, the radiobiological sensitivity to dose fractionation for late‐responding tissues, such as the spinal cord and brainstem, makes it even more critical to accurately predict doses to be delivered to these critical organs at risk when hypofractionation treatment schemes are employed.

Stereotactic radiosurgery has been used for the treatment of skull base tumors over the past few decades and has demonstrated durable tumor control and symptomatic relief with acceptable toxicity in the patients with malignant tumors less than 4 cm in diameter.^18^, ^19^ Many researchers have described the utilization of SRT for reirradiation of head and neck tumors, primarily using CyberKnife (Accuray Inc., Sunnyvale, CA) radiosurgery^20^, ^21^, ^22^, ^23^, ^24^ and linac‐based SRT.^25^, ^26^ Specifically, Unger and colleagues^(20)^ presented a feasibility study reirradiating head and neck cancer patients using CyberKnife technique with fractionated‐SRS scheme. For 65 patients, the median initial radiation dose was 67 Gy (in 33 fractions), and the median reirradiation SRS dose was 30 Gy (21‐35 Gy) in 2‐5 fractions. In their study, the median follow‐up for surviving patients was 16 months. Out of 56 patients who were evaluated for response, 30 (54%) patients had complete response. However, seven patients (11%) experienced severe reirradiation‐related toxicity. Similarly, Siddiqui and colleagues^(26)^ studied the feasibility, safety, and efficacy of linac‐based SBRT in patients with primary, recurrent, and metastatic head and neck tumors.

Fifty‐five tumors in 44 patients were treated with either a single fraction of 13‐18 Gy or 5‐8 fraction schedules to a total dose of 36‐48 Gy. For the 24 patients who had follow‐up scans, the percentage of reduction in tumor volume was 52%±38%. Local control rate at one year was 83.3% for primary tumors and 60.6% for recurrent tumors. The University of Pittsburgh experience suggests a dose response with a trend toward improved survival with doses of 40 Gy or higher.^(27)^ However, none of these studies reported Monte Carlo‐computed dose distributions for the head and neck SRT patients.

Our retrospective study demonstrated the feasibility of using Monte Carlo‐based treatment planning and dose calculation for clinical head and neck SRT patients. Based upon our experiences and previously XVMC validated literature, XVMC appears to more accurately predict dose distributions in areas of lower density and sites of increased tissue heterogeneity. As a result, while employing large daily fraction size doses for head and neck cancer patients treated with SRT, accuracy of both patient setup and dose calculation are of critical importance. This is even more important in the case of previous irradiation, in which the ability to provide an acceptable dose coverage is limited by the proximity of radiation tolerances of adjacent OARs. In the future, we plan to follow up these patients to prospectively review local control for those who received non‐Monte Carlo computed SRT dose (PB‐hete algorithm) to the target volume in order to further study its potential radiobiological effects on late effects to the organs at risk. We are also planning to implement XVMC calculations for the head and neck SRT patients clinically.

## V. CONCLUSIONS

Our results demonstrate that Monte Carlo‐based method, such as XVMC algorithm in iPlan, can be used clinically to predict more accurate dose distributions in the presence of inhomogeneity typical for head and neck SRT patients. Due to the lack of lateral electronic equilibrium, PB‐hete algorithms may not be able to accurately predict head and neck SRT dose calculations. The lower value of $V_{p}$ up to 7.8% and higher OARs doses demonstrated that targets may potentially be underdosed and critical OARs could get overdosed when utilizing PB‐hete dose calculation compared to XVMC. The PB‐hete algorithm may not be sufficiently accurate for head and neck SRT treatment planning, despite the fact that not all the dosimetric parameters were statistically significant. Our preliminary results suggest that Monte Carlo‐based dose calculation may be the method of choice for relatively smaller field dosimetry such as head and neck SRT — especially in cases of reirradiation when accurate dose calculation is critical. Special attention may need to be applied to the planning algorithm used while evaluating for the target coverage and OARs doses when planning for head and neck SRT based on tumor size and location especially adjacent to heterogeneous tissues.

## COPYRIGHT

This work is licensed under a Creative Commons Attribution 4.0 International License.

## References

[1] Benedict SH , Yenice KM , Followill D , et al. Stereotactic body radiation therapy: the report of AAPM Task Group 101. Med Phys. 2010;37(8):4078–100. 20879569 10.1118/1.3438081

[2] Mohan R , Chui C , Lidofsky L . Differential pencil beam dose computation model for photons. Med Phys. 1986;13(1):64–73. 3951411 10.1118/1.595924

[3] Mackie TR , Scrimger JW , Battista JJ . A convolution method of calculating dose for 15‐MV x rays. Med Phys. 1985;12(2):188–96. 4000075 10.1118/1.595774

[4] Wang L , Yorke E , Chui CS . Monte Carlo evaluation of tissue inhomogeneity effects in the treatment of the head and neck. Int J Radiat Oncol Biol Phys. 2001;50(5):1339–49. 11483347 10.1016/s0360-3016(01)01614-5

[5] Solberg TD , Holly FE , De Salles AA , Wallace RE , Smathers JB . Implications of tissues heterogeneity for radiosurgery in head and neck tumors. Int J Radiat Oncol Biol Phys. 1995;32(1):235–39. 7721621 10.1016/0360-3016(94)00495-7

[6] Wang L , Chui CS , Lovelock M . A patient‐specific Monte Carlo dose‐calculation method for photon beams. Med Phys. 1998;25(6):867–78. 9650174 10.1118/1.598262

[7] Wang L , Lovelock M , Chui CS . Experimental verification of a CT‐based Monte Carlo dose‐calculation method in heterogeneous phantoms. Med Phys. 1999;26(12):2626–34. 10619248 10.1118/1.598802

[8] Fippel M . Fast Monte Carlo dose calculation for photon beams based on the VMC electron algorithm. Med Phys. 1999;26(8):1466–75. 10501045 10.1118/1.598676

[9] Fippel M , Laub W , Huber B , Nüsslin F . Experimental investigation of a fast Monte Carlo photon beam dose calculation algorithm. Phys Med Biol. 1999;44(12):3039–54. 10616153 10.1088/0031-9155/44/12/313

[10] Brainlab Physics . Technical reference guide. Revision 1.2. Feldkirchen, Germany: Brainlab AG; 2010.

[11] Sethi A , Leo P , Kabat C , Cecilio P . Validation of Monte Carlo dose algorithm in heterogeneous medium [abstract]. Med Phys. 2013;40(6):329.

[12] Fragoso M , Wen N , Kumar S , et al. Dosimetric verification and clinical evaluation of a new commercially available Monte Carlo‐based dose algorithm for application in stereotactic body radiation therapy (SBRT) treatment planning. Phys Med Biol. 2010;55(16):4445–64. 20668343 10.1088/0031-9155/55/16/S02

[13] Petoukhova AL , van Wingerden K , Wiggenraad RG , et al. Verification measurements and clinical evaluation of the iPlan RT Monte Carlo dose algorithm for 6MV photon energy. Phys Med Biol. 2010;55(16):4601–14. 20668337 10.1088/0031-9155/55/16/S13

[14] Petoukhova AL , Wiggenraad RG , van de Vaart PJM , et al. Evaluation and implementation of iPlan RT Monte Carlo dose algorithm for head and neck and lung cancer patients. Int J Radiat Oncol Biol Phys. 2009;79(3):S674

[15] Badkul R , Pokhrel D , Jiang H , Wang F , Kumar P . Dosimetric evaluation and clinical implementation of iPlan Monte Carlo algorithm for lung stereotactic ablative radiotherapy (SABR) [abstract]. Med Phys. 2013;40(6):300. 10.1120/jacmp.v16i1.5058 PMC5689968 25679161

[16] Estes C , Pokhrel D , Kimler BF , et al. Comparative analysis of Monte Carlo and pencil beam algorithm‐derived dose distributions for tumors and normal tissue in lung stereotactic ablative radiation therapy. Int J Radiat Oncol Biol Phys. 2014;90(1):S905.

[17] Pokhrel D , Badkul R , Jiang H , et al. Dosimetric evaluation of centrally located lung tumors: a Monte Carlo (MC) study of lung SBRT planning. Med Phys. 2014;41(6):167.

[18] Miller RC , Foote RL , Coffey RJ , et al. The role of stereotactic radiosurgery in the treatment of malignant skull base tumors. Int J Radiat Oncol Biol Phys. 1997;39(5):977–81. 9392534 10.1016/s0360-3016(97)00377-5

[19] Cmelak AJ , Cox RS , Adler JR , Fee WE Jr , Goffinet DR . Radiosurgery for skull base malignances and nasopharyngeal carcinoma. Int J Radiat Oncol Biol Phys 1997;37(5):997–1003 9169805 10.1016/s0360-3016(97)00111-9

[20] Unger KR , Lominska CE , Deeken JF , et al. Fractionated stereotactic radiosurgery for reirradiation of head‐and‐neck cancer. Int J Radiat Oncol Biol Phys. 2010;77(5):1411–19. 20056341 10.1016/j.ijrobp.2009.06.070

[21] Roh KW , Jang JS , Kim MS , et al. Fractionated stereotactic radiotherapy as reirradiation for locally recurrent head and neck cancer. Int J Radiat Oncol Biol Phys. 2009;74(5):1348–55. 19117695 10.1016/j.ijrobp.2008.10.013

[22] Voynov G , Heron DE , Burton S , et al. Frameless stereotactic radiosurgery for recurrent head and neck carcinoma. Technol Cancer Res Treat. 2006;5(5):529–35. 16981796 10.1177/153303460600500510

[23] Cengiz M , Özyi$gbit G, Yazici G , et al. Salvage reirradiation with stereotactic body radiotherapy for locally recurrent head‐and‐neck tumors. Int J Radiat Oncol Biol Phys. 2011;81(1):104–09. 20675075 10.1016/j.ijrobp.2010.04.027

[24] Comet B , Kramar A , Faivre‐Pierret M , et al. Salvage stereotactic reirradiation with or without cetuximab for locally recurrent head‐and‐neck cancer: a feasibility study. Int J Radiat Oncol Biol Phys. 2012;84(1):203–09. 22331006 10.1016/j.ijrobp.2011.11.054

[25] Ryu S , Khan M , Yin FF , et al. Image‐guided radiosurgery of head and neck cancers. Otolaryngol Head Neck Surg. 2004;130(6):690–97. 15195054 10.1016/j.otohns.2003.10.009

[26] Siddiqui F , Patel M , Khan M , et al. Stereotactic body radiation therapy for primary, recurrent, and metastatic tumors in the head‐and‐neck region. Int J Radiat Oncol Biol Phys. 2009;74(4):1047–53. 19327895 10.1016/j.ijrobp.2008.09.022

[27] Rwigema J‐CM , Heron DE , Ferris RL , et al. The impact of tumor volume and radiotherapy dose on outcome in previously irradiated recurrent squamous cell carcinoma of the head and neck treated with stereotactic body radiation therapy. Am J Clin Oncol. 2011;34(4):372–79. 20859194 10.1097/COC.0b013e3181e84dc0 PMC3177149

